# *Helicobacter pylori* Infection and Upper Gastrointestinal Disorders

**DOI:** 10.1155/2013/896209

**Published:** 2013-07-30

**Authors:** Vikram Kate, Nanda K. Maroju, N. Ananthakrishnan

**Affiliations:** ^1^Department of General and Gastrointestinal Surgery, Jawaharlal Institute of Postgraduate Medical Education and Research, Pondicherry 605006, India; ^2^Jawaharlal Institute of Postgraduate Medical Education and Research, Pondicherry 605006, India; ^3^Mahatma Gandhi Medical College & Research Institute, Pondicherry 607402, India

It has been thirty years since the two Australians Robin Warren and Barry Marshall discovered *Helicobacter pylori* (*H. pylori*) in 1983 [1]. In order to fulfil the Koch's postulates, Marshall and Morris drank a solution which was a suspension of *H. pylori.* This produced gastritis from which the bacteria could be reisolated [2]. It will be interesting to see how the approach to treatment has progressed in these three decades after the discovery of the organism-based both on consensus guidelines and other research in this field. The changes in the consensus statements have been highlighted. The isolation of *H. pylori* from the gastric mucosa and the report of the organism's urease activity generated excitement especially when it was postulated by Marshall that these microorganisms could be the cause of gastritis and could be a dominant etiological factor in the pathogenesis of peptic ulcer disease (PUD). With the isolation of *H. pylori*, floodgates opened to a new era of discovery and understanding of gastroduodenal pathology. These results were a paradigm shift from the earlier belief that PUD disease was related to stress, lifestyle, and acid secretion based on the dictum of Schwarz “no acid no ulcer.” The early nineteen eighties when Warren and Marshall reported their findings coincided with omeprazole belonging to the group of proton pump inhibitors (PPIs) being introduced. This PPI was documented as a potent antisecretory agent which yielded very good results for ulcer healing and achieving a potential cure for patients with PUD when compared to the earlier drugs belonging to the group of H_2_ receptor antagonists. Hence there was lot of scepticism in the gastroenterology community world over to accept that PUD was the result of infection. However, it was found that patients with PUD continued to have remission of the disease even after cessation of antisecretory therapy.

The knowledge about *H. pylori* continued to evolve at a rapid rate, and in 1994 the first guidelines on treatment of infection with this organism were published by the US National Institute of Health (NIH) in JAMA [3]. The consensus statement was that patients with PUD and *H. pylori* infection require eradication of *H. pylori* and antisecretory drugs whether on first presentation or on recurrence of the disease, whereas in patients with nonulcer dyspepsia (NUD) with *H. pylori* infection the value of treating *H. pylori* infection remained to be determined. It was also suggested in the consensus statement that the relationship between *H. pylori* infection and gastric cancers required further exploration.

The discrepancy in the treatment protocols in different countries and lack of national guidelines called for another meeting of The European *Helicobacter pylori* Study Group (EHPSG) at Maastricht, The Netherlands, in 1996, to form guidelines for general health care issues pertaining to *H. pylori* infection [4]. In addition to the recommendations of the NIH, treatment was strongly recommended for patients with low-grade gastric-mucosa-associated lymphoid tissue (MALT) lymphoma, bleeding PUD, gastritis with severe abnormalities, and following early resection for gastric cancer. It was also advised that eradication was advisable in patients less than 45 years of age with functional dyspepsia in whom alarm symptoms were ruled out. The Maastricht guidelines advised noninvasive tests such as the urea breath test (UBT) or serology for diagnosis before therapy. Eradication was also advised in first degree relatives of gastric cancer patients, planned or existing (nonsteroidal anti-inflammatory drug) NSAID therapy, and following surgery for peptic ulcer. The seven-day standard triple therapy comprising PPI with two antibiotics was recommended in supersession of the previous classical bismuth triple therapy as it was found to be superior in efficacy, with fewer side effects and better compliance. An eradication rate of over 80% on intention-to-treat (ITT) basis was considered satisfactory.

In the next four years, significant progress was made in different aspects of *H. pylori*-associated disease which necessitated the second meeting of the EHPSG to update the previous guidelines [5]. This meeting of the experts was held again at Maastricht in the year 2000. All the strongly recommended indications for *H. pylori* eradication from the earlier Maastricht guidelines were reinforced in this meeting. In patients with PUD, it was recommended that this should include active and inactive disease, complicated disease, and also following surgery for peptic ulcer. The importance of eradication of *H. pylori* was stressed again in first degree patients with gastric cancer and patients with functional dyspepsia. The benefit in patients with functional dyspepsia was limited and was seen in less than 10% of patients; however, this was comparable to other treatments with antisecretory or antinociceptive drugs [6, 7]. It was stated that there was strong evidence that eradication of *H. pylori* is not associated with the development of gastroesophageal disease (GERD) in most cases nor does it exacerbate GERD [8, 9]. The guidelines stated that it was advisable to eradicate *H. pylori* in patients with GERD needing long-term profound acid suppression. The meeting also concluded that *H. pylori* and NSAID use are independent factors for PUD. *H. pylori* eradication was not indicated in extra-alimentary tract disorders. A modification on the Maastricht I report was that ranitidine bismuth citrate (RBC) combined with clarithromycin, amoxicillin, or metronidazole was included as first line triple therapy as it demonstrated similar efficacy to standard triple therapy where a PPI replaced RBC combined with two antibiotics [10]. A seven-day standard triple therapy was recommended. Quadruple therapy comprising a PPI, bismuth, metronidazole, and tetracycline was recommended as second-line therapy. Confirmation of eradication was advised at the end of 4 weeks following therapy by using non-invasive tests like UBT or stool antigen test (SAT) when endoscopy was not indicated for assessing the primary disorder. Serology was considered as an inappropriate method to determine eradication.

The Third Maastricht Consensus Conference convened to update guidelines on the management of *H. pylori* infection was held in Maastricht in 2005 [11]. The modified recommendations of this meeting included for the first time eradication of *H. pylori* in patients with extraintestinal diseases such as iron-deficiency anemia (IDA) and immune thrombocytopenic purpura (ITP) based on the reversal of IDA and significant positive platelet increase in patients with ITP [12, 13]. *H. pylori *was not found to have any proven role in other extraintestinal disorders. Eradication was recommended in naive NSAID users as it was likely to prevent peptic ulcer and bleeding. However, in patients receiving long-term NSAIDs with peptic ulcer or peptic ulcer bleeding, PPI maintenance treatment was considered superior to *H. pylori *eradication therapy in preventing ulcer recurrence or bleeding. Contrary to previous opinion it was found that there is a negative association between the prevalence of *H. pylori* and GERD especially with CagA positive strains as the incidence of Barrett's esophagus and gastroesophageal (GE) was reported to be lower in them [14]. However, it was reemphasized that eradication of *H. pylori* does not exacerbate GERD either when left untreated or when patients are being treated with PPI for GERD [11]. The duration for standard triple therapy was increased to 14 days. This was considered superior to seven-day therapy unless local studies showed that seven-day therapy was effective [15]. In areas with low metronidazole resistance (<40%), the combination of PPI-clarithromycin-metronidazole was superior to PPI-clarithromycin-amoxicillin independent of the sensitivity to clarithromycin [16]. SAT using monoclonal antibodies was preferable to polyclonal antibody test. A laboratory-based test was superior to office-based test. 

The most recent fourth conference in this series was held in Florence in 2010. However, the Maastricht methodology was used for updating of the guidelines for *H. pylori* treatment. Hence it was termed as the Maastricht/Florence consensus report [17]. It was mentioned that patients who are on long-term PPIs with *H. pylori* infection are associated with corpus-predominant gastritis which can progress to atrophic gastritis. In these patients, eradication of *H. pylori* heals the gastritis and prevents progression to atrophic gastritis [18]. Apart from IDA and ITP, the other extraintestinal disorder in which eradication of *H. pylori* was recommended was Vitamin B12 deficiency. It was reinforced that SATs by a laboratory method using monoclonal antibodies should be used as an alternative to UBT as a non-invasive method to detect eradication of *H. pylori* [19, 20]. In areas of high clarithromycin resistance (more than 15–20%), bismuth quadruple therapy was recommended. If bismuth-based therapy was not available, then nonbismuth quadruple therapy or sequential therapy was indicated [21, 22]. *H. pylori* eradication to prevent gastric cancer was recommended in populations at high risk. This was in contradistinction to earlier guidelines which recommended eradication only after gastrectomy for gastric surgery. Eradication was also recommended in patients with risk of severe pan-gastritis, corpus predominant gastritis, or severe atrophy. Patients with strong environmental risk factors like heavy smokers or who have high exposure to dust, coal, cement, or working in quarries should have therapy for *H. pylori*.

It is interesting to see the gradual change in the recommendations for diagnosis and treatment of *H. pylori* infection over these years and the appearance of extraintestinal diseases as an indication for eradication. However, more and more diseases continue to be attributed to infection with this organism or are shown to be associated with infection with this organism such as inflammatory bowel disease or colonic carcinoma. We will have to wait till future studies throw some light on the causal association of this organism with other extra-alimentary diseases. 

*Vikram Kate* *Vikram Kate* *Nanda K. Maroju* *Nanda K. Maroju* *N. Ananthakrishnan* *N. Ananthakrishnan*
